# Leclercia adecarboxylata: A Rare and Emerging Cause of Gram-Negative Infective Endocarditis

**DOI:** 10.7759/cureus.103924

**Published:** 2026-02-19

**Authors:** Omar Al-Shujairi, Vino Srirathan, Mohammad Khadeem Rumjaun, Fiona Price

**Affiliations:** 1 Cardiology, Northern Care Alliance NHS Foundation Trust - Salford Care Organization, Salford, GBR; 2 Microbiology, Northern Care Alliance NHS Foundation Trust - Salford Care Organization, Salford, GBR; 3 Microbiology, Manchester University NHS Foundation Trust, Manchester, GBR

**Keywords:** aortic valve infective endocarditis, aortic valve vegetations, gram-negative endocarditis, gram-negative sepsis, infective endocarditis, leclercia adecarboxylata, sepsis in liver cirrhosis, very rare cause of endocarditis

## Abstract

Infective endocarditis (IE) is a serious and life-threatening condition often associated with high morbidity and mortality, particularly in immunocompromised populations. We present a case of IE caused by the gram-negative pathogen *Leclercia* *adecarboxylata* in a 37-year-old male with advanced alcohol-related liver cirrhosis complicated by portal hypertension, ascites, and episodes of hepatic encephalopathy. The patient presented initially with decompensation of his liver disease and later developed a fever. *L.* *adecarboxylata* was identified in blood cultures together with an aortic valve lesion. Despite aggressive management with a prolonged course of intravenous antibiotics and supportive care, the patient's clinical course was marked by repeated bacteremia and progressive decompensation of liver disease. This case illustrates the increasing clinical significance of *L.* *adecarboxylata* in immunocompromised hosts and highlights the unique challenges of managing IE in a patient with cirrhosis.

## Introduction

Infective endocarditis (IE) remains a serious and life-threatening condition often associated with high morbidity and mortality, particularly in immunocompromised populations. Gram-positive organisms such as Staphylococcus and Streptococcus species are the most common causes of IE, while gram-negative pathogens rarely cause it [1].

To date, only four cases of L. adecarboxylata IE have been published [2-5]. The case presented here highlights the clinical course, challenges, and outcomes of managing a rare case of L. adecarboxylata IE in a cirrhotic patient of which clinicians should be aware.

L. adecarboxylata is a motile, gram-negative bacillus in the Enterobacteriaceae family that is a rarely isolated cause of disease, despite being ubiquitous in nature [5].

## Case presentation

We present the case of a 37-year-old man with a medical history of advanced alcohol-related liver disease complicated by portal hypertension with gastropathy, esophageal varices, and ascites. He presented to the hospital in March 2024 with lethargy and difficulty mobilizing resulting from a fall and long lie of seven hours. He had had difficulty sleeping over the previous four days, had felt delirious, and was suffering auditory and visual hallucinations. He had been discharged from a hospital one week earlier and had restarted drinking alcohol for 48 hours before stopping again. Over the previous 12 months, he had been in and out of the hospital on numerous occasions with a variety of presentations, including rectal hemorrhoidal bleeding, alcohol withdrawal, hepatic encephalopathy, low mood, and suicidal ideation. He had also developed peripheral edema, including scrotal swelling, over a nine-month period coinciding with a phimosis abnormality.

**Figure 1:**
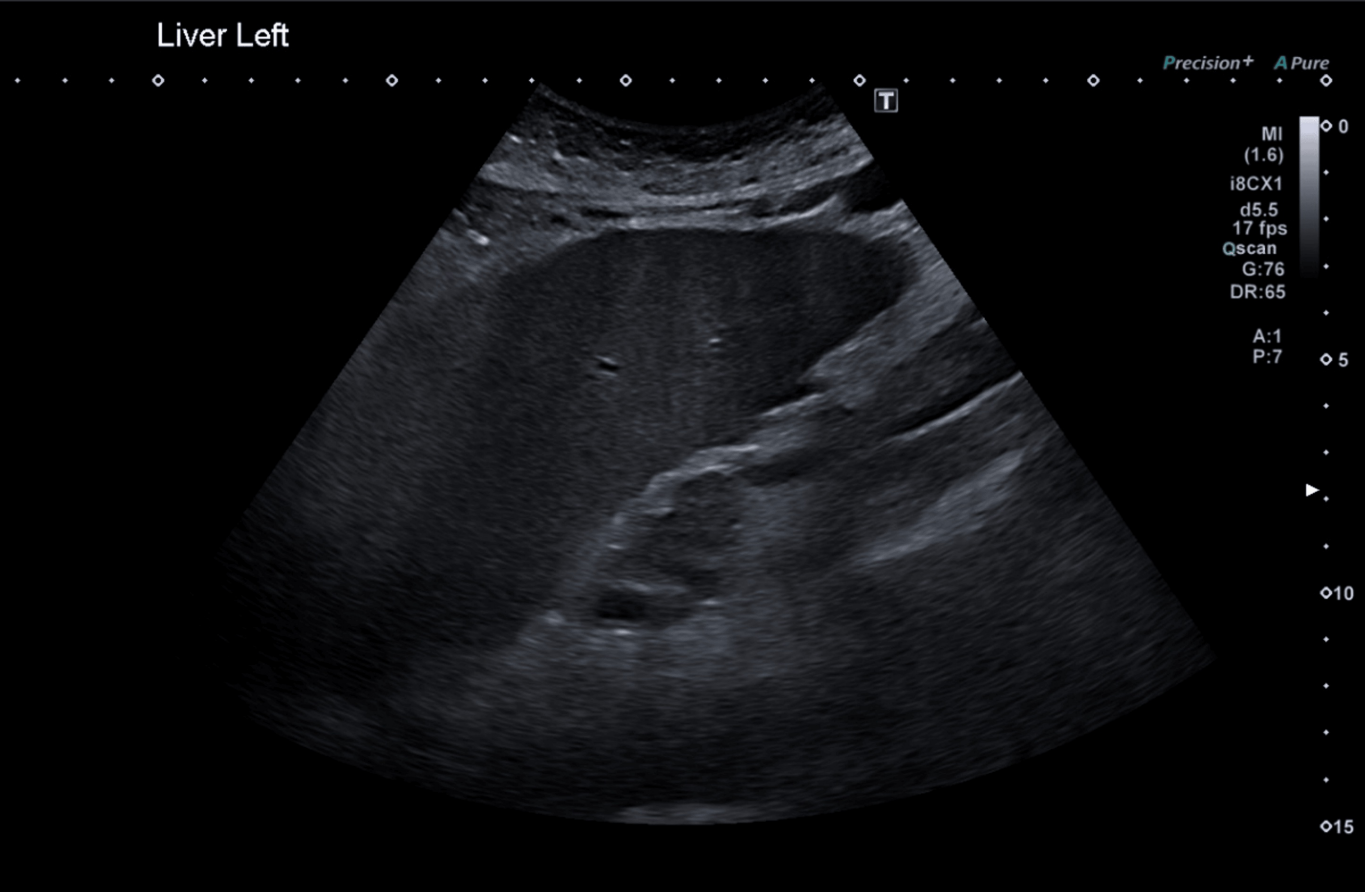
Ultrasound showing moderately enlarged cirrhotic liver and a thick-walled gallbladder without calculi or sludge.

A liver ultrasound six weeks before this presentation, shown in Figure 1, confirmed a moderately enlarged cirrhotic liver, slight ascites around the spleen, and a thick-walled gallbladder without calculi or sludge. The patient’s bilirubin had been elevated since his first presentation to our hospital in late 2022, and, since 2023, had remained above 100 micromol/L. On this presentation to the hospital, his blood tests were as shown in Table 1.

**Table 1:** Patient’s blood tests. L, liter; micromol, micromoles; g, gram; PT, prothrombin time.

Blood test	Current level	Normal reference range
Bilirubin	168 micromol/L	0-20 micromol/L
Albumin	37 g/L	35-50 g/L
PT	23.7 seconds	9.7-11.8 seconds

On admission, the patient was oriented to time, person, and place. He was visibly jaundiced and did not have liver flap. His abdomen was soft and without ascites on clinical examination, and he had peripheral lower-body edema to the level of the mid-abdomen. He was hemodynamically stable, and his heart examination was normal on auscultation as well. His level of hepatic encephalopathy was graded as 1-2 at the time of presentation. This finding confirmed his Child-Pugh C grading (total: 13 points). No consolidation was seen on the admission chest X-ray, but there were signs of perihilar fluid overload.

A dose of the diuretic furosemide was uptitrated to 40 mg twice daily, and spironolactone was continued for the fluid overload. Coagulopathy was corrected with 10 mg vitamin K daily over three days. Within a few days, the patient developed an acute kidney injury, resulting in the cessation of his diuretics and the initiation of albumin infusion. One week after admission, he was found to be febrile, with a tympanic temperature of 39.4°C, and to have tachycardia accompanied by a non-productive cough and shortness of breath. A full septic screen was taken, including blood cultures and ascitic tap, and empiric IV piperacillin-tazobactam was started. No new consolidation was observed on a repeat chest X-ray.

Two sets of blood cultures were performed, one of which identified gram-negative rods that matrix-assisted laser desorption ionization-time of flight spectrometry confirmed to be L. adecarboxylata. Disc diffusion antimicrobial sensitivity testing indicated that this organism was sensitive to all first-line gram-negative antibiotics, including ampicillin, co-amoxiclav, piperacillin-tazobactam, gentamicin, ciprofloxacin, and co-trimoxazole. The patient’s ascitic fluid had a white cell count of 4, and no organisms were seen on microscopy. Enrichment of ascites later identified gram-positive bacilli and cultured Dietzia species, but this finding was not considered clinically relevant. Piperacillin-tazobactam was continued for a week, and the patient improved clinically.

**Figure 2:**
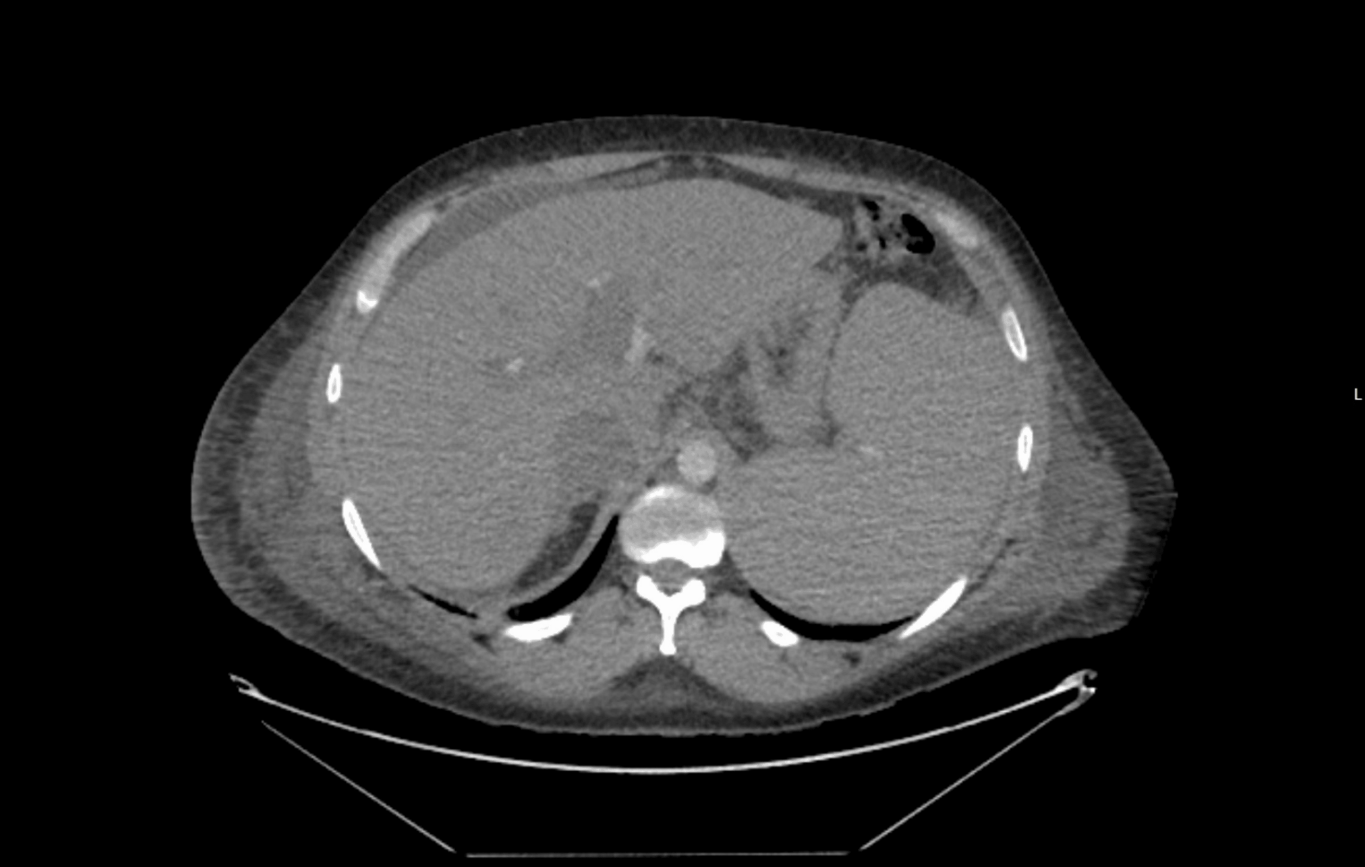
Computerized tomography of the thorax, abdomen, and pelvis showing enlarged liver with cirrhotic changes.

**Figure 3:**
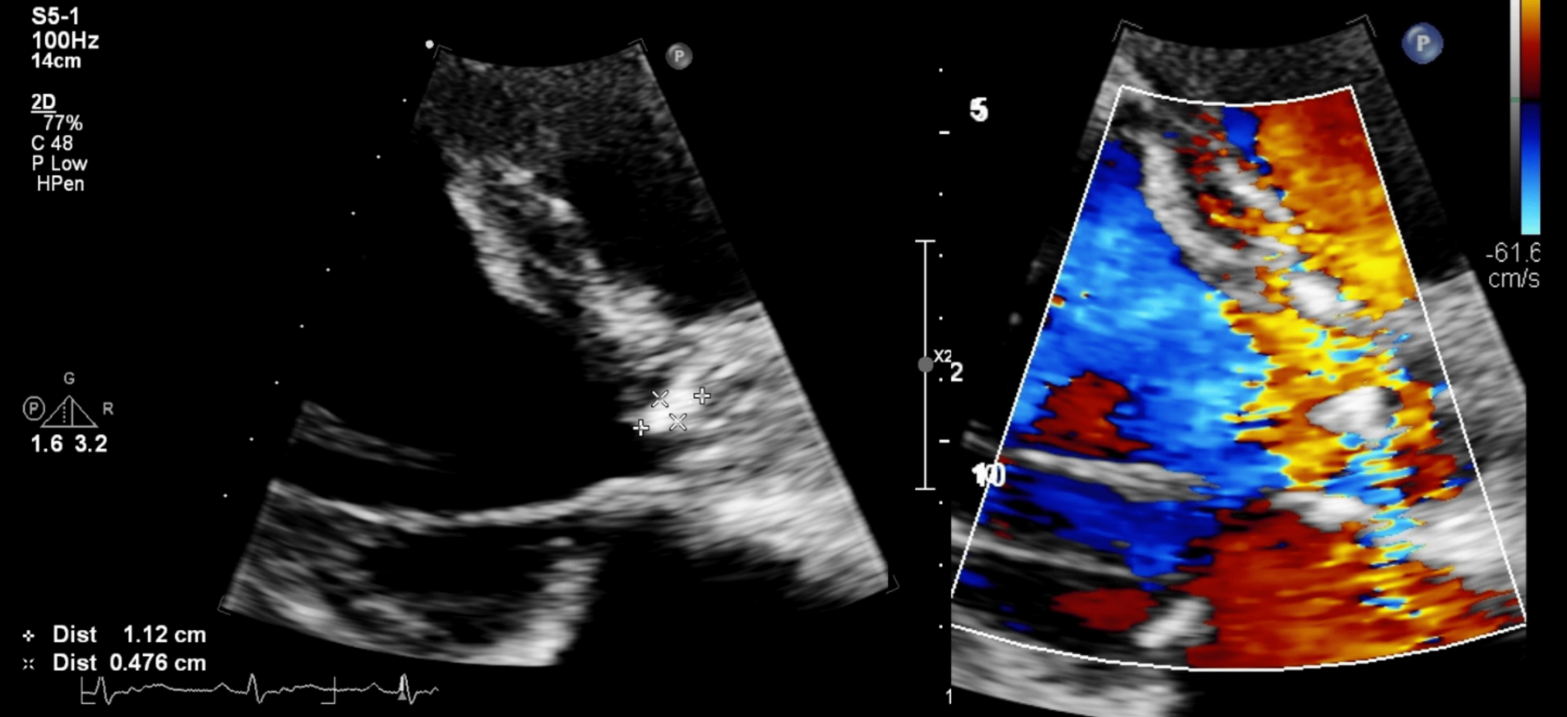
Echocardiogram showing aortic valve vegetation and moderate aortic regurgitation.

One week after the antibiotic treatment, the patient was again febrile and tachycardic. This change in his condition coincided with a flare-up of pain in the scrotum. On examination, ongoing scrotal edema without redness or skin change was observed. Piperacillin-tazobactam was recommenced. A repeat blood culture (one sample was sent) was again positive for L. adecarboxylata, and this finding prompted further investigation with computerized tomography (CT) of the thorax, abdomen, and pelvis (Figure 2) and an echocardiogram. The CT scan revealed bilateral pulmonary ground glass opacities suggestive of infection, mild diffuse ascites, and mesenteric lymphadenopathy together with marked splenomegaly. Ultrasound of the testes raised suspicion of a right-sided testicular abscess. Urine culture at the time of blood culture grew a sensitive strain of Klebsiella oxytoca resistant only to amoxicillin. The echocardiogram reported a small echogenic mass attached to the aortic valve highly suspicious of vegetation with moderate aortic regurgitation (Figure 3).

When thinking about the modified Duke criteria for IE, our patient met one major criterion, which is evident in the echocardiogram finding of a vegetation. Also, he met two minor criteria in fever and microbiological evidence, as he had two positive blood cultures with an atypical microorganism. This would make IE a possible diagnosis.

The case was discussed during a meeting of the regional IE multidisciplinary team, and the patient was recommended for medical management but not for surgical intervention because of his advanced liver cirrhosis. Also, the team considered it unsafe for the patient to undergo a transesophageal echocardiogram given the high risk of bleeding from the esophageal varices. A plan was made to complete a six-week course of intravenous piperacillin-tazobactam. Despite all guidelines recommending combination therapy, the decision was for a single agent with piperacillin-tazobactam, as the organism was sensitive on two separate blood culture sets.

The patient responded very well clinically, and a new set of blood cultures was negative, confirming that 31 days of intravenous piperacillin-tazobactam did help clear the L. adecarboxylata. He was about to be discharged home but, four days after stopping the antibiotic treatment, was again febrile, so piperacillin-tazobactam was restarted. Repeat blood cultures initially found Corynebacterium striatum in two sets, so intravenous vancomycin was added to the antibiotic treatment.

**Figure 4:**
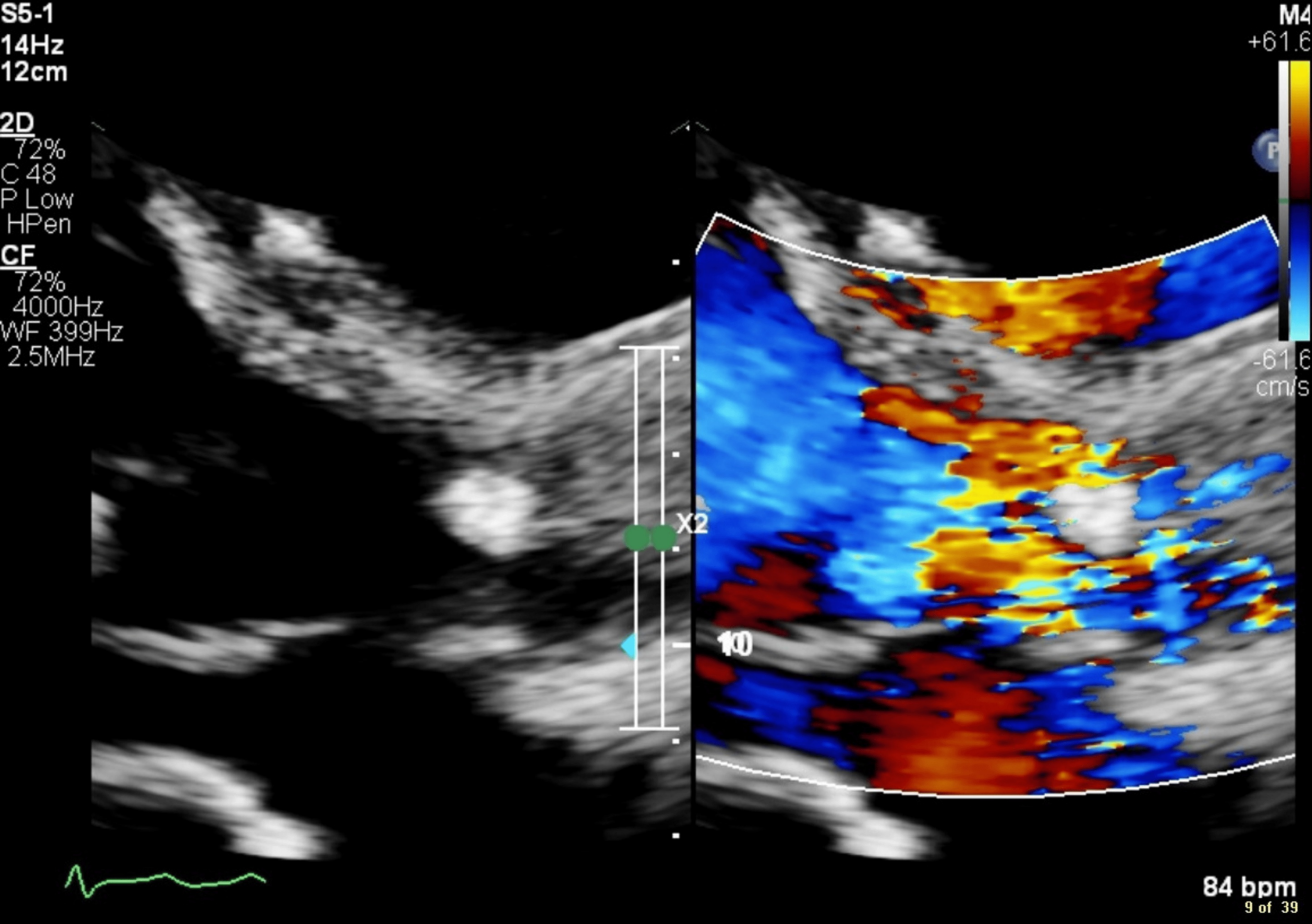
Repeat echocardiogram showing the aortic valve vegetation

One week later, a further repeat blood culture detected Escherichia coli that, on antimicrobial sensitivity testing, was found phenotypically to be carrying extended-spectrum beta-lactamase genes. An echocardiogram was repeated at this stage, which again showed the aortic valve vegetations (Figure 4). The vegetations were reported to be less mobile than before.

**Figure 5:**
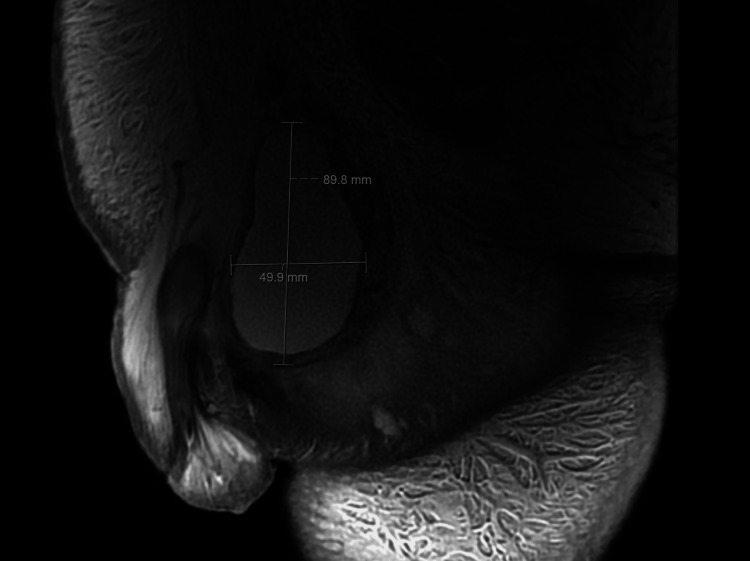
Magnetic resonance scan of the genital tract showing loculated fluid collection.

A repeat groin ultrasound again reported a left-sided hydrocele and a 6.6 x 9.1 x 7.1 mm abscess at the base of the penis. However, a magnetic resonance scan of the genital tract (Figure 5) excluded abscess in the penis and instead indicated that the left testicular changes involved a loculated fluid collection measuring 6.0 x 5.5 x 9.0 cm rather than a hydrocele. The patient was deemed not well enough for surgical intervention to treat this condition, and he did not undergo diagnostic aspiration of the left testis.

The patient continued to have pain and progressive lower extremity edema that the management of his hepatorenal syndrome did not reverse. The repeated infections were considered a consequence of progressive multiorgan failure, so the decision was made to focus on comfort care. His care was transitioned to focus on palliation, and he passed away comfortably a few days later.

## Discussion

The organisms most often recognized as the cause of gram-negative IE are known as the HACEK group, consisting of Haemophilus spp., Aggregatibacter actinomycetemcomitans, Cardiobacterium hominis, Eikenella corrodens, and Kingella spp. IE caused by non-HACEK gram-negative bacilli is very rare and associated with high mortality [1]. The risk factors for non-HACEK gram-negative IE include the presence of prosthetic material [1,6] and being a person who injects drugs [7,8].

The usual species found in cases of non-HACEK gram-negative IE include Escherichia coli and Pseudomonas [1,6]. Endocarditis caused by L. adecarboxylata is exceedingly rare. This gram-negative rod-shaped bacterium is commonly found in water and soil and also exists in the intestinal flora of some animals. Infections by L. adecarboxylata have been underestimated and underreported for decades, mainly because most strains are misidentified, often as E. coli, with which L. adecarboxylata shares a number of metabolic and morphological specifications. The organism has been isolated in cases of polymicrobial infections, urinary tract infections, pneumonia, and catheter-related bloodstream infections. Hepatic cirrhosis has been reported as a risk factor [9]. Historically regarded as a non-pathogenic environmental organism, L. adecarboxylata is now recognized as an emerging opportunistic pathogen [10].

Only four case reports of IE caused by monomicrobial infection with L. adecarboxylata have been reported [2-5]. Three were immunocompetent hosts, and one of the patients was immunocompromised and had a background of cancer.

The source of gram-negative IE is often presumed to be the genitourinary tract [6,11]. Our patient was known to have lower-body and scrotal edema and left testicular fluid collection. Since culture of this fluid was not performed, it is not possible to confirm that it was the source. He also had a history of positive urine cultures, having grown Klebsiella oxytoca on a urine culture during this admission to the hospital and Enterococcus faecalis on previous recent admissions. The same authors report immunosuppression as being a risk factor [6,11]. Underlying liver cirrhosis was the immunosuppressive condition that predisposed our patient to this rare infection. The bacterial etiology of endocarditis in cirrhotic patients is comparable to that in the general population [12], but the former may be at increased risk of infection caused by bacterial translocation from the gastrointestinal tract as a result of altered intestinal permeability.

Other potential sources of infection for gram-negative endocarditis include indwelling vascular lines and the hospital environment, particularly in the case of non-fermenting organisms such as Pseudomonas [11].

Other than peripheral venous cannulas and a peripherally inserted central catheter line inserted after the onset of bacteremia, our patient did not have any long-term prosthetic devices or a history of urinary catheterization preceding this illness.

The consensus guidelines for the treatment of gram-negative IE from the American Heart Association (AHA), the Infectious Disease Society of America (IDSA), and the European Society of Cardiology recommend the use of beta-lactam in combination with a second agent. In our patient’s case, there are no clinical breakpoints to interpret the susceptibility of L. adecarboxylata because of the rarity of this organism, so the optimal therapy was not known. A recent single-center cohort study found no difference in the composite outcomes between patients treated with monotherapy and those who received combination therapy [13]. The AHA and IDSA guidelines also recommend early consideration of valve surgery, which was not possible for our patient because of his advanced liver disease and cirrhosis. Cirrhosis has been shown to be an independent risk factor for mortality in endocarditis [12], and this risk is related to the level of liver dysfunction. Our patient’s Childs-Pugh grade of C placed him in the high-risk group.

We did not treat the patient with aminoglycoside as a second agent because of the risk of nephrotoxicity and hepatorenal syndrome. He was subsequently found to have bacteremia with Corynebacterium and E. coli, the source of which was unclear. Rather than fully investigating and treating the additional bacteremia, we decided to focus on comfort care.

We faced multiple limitations in our case study. It was not possible in our patient's case to perform a transesophageal echocardiogram, which would have given a better clinical picture. Also, there were multiple possible foci of infection, and despite that these other possible sources were ruled out clinically, no investigations were carried out to control the source of infection. One final limitation that we faced, although not common in clinical practice, is the fact that we don't have histopathological confirmation of our diagnosis and that is one of the major criteria according to modified Duke criteria.

## Conclusions

Gram-negative endocarditis is rare and has a high rate of morbidity and mortality. We highlight a rare presentation of L. adecarboxylata endocarditis diagnosed by recurrent bacteremia and aortic valve vegetation. Our patient was an immunocompromised host, and the origin of the infection remained unconfirmed but may have involved translocation from the genitourinary or gastrointestinal tract. Because of the patient’s background of cirrhosis, surgical management was not feasible, and, unfortunately, prolonged antibiotic treatment did not clear the infection.
